# CTGF promotes the repair and regeneration of alveoli after acute lung injury by promoting the proliferation of subpopulation of AEC2s

**DOI:** 10.1186/s12931-023-02512-4

**Published:** 2023-09-23

**Authors:** Jianhui Sun, Huacai Zhang, Di Liu, Wenyi Liu, Juan Du, Dalin Wen, Luoxi Li, Anqiang Zhang, Jianxin Jiang, Ling Zeng

**Affiliations:** Department of Trauma Medical Center, Daping Hospital, State Key Laboratory of Trauma, Burns and Combined Injury, Army Medical University, Chongqing, 400042 China

**Keywords:** Acute lung injury, Acute respiratory distress syndrome, Alveolar regeneration, Connective tissue growth factor, Alveolar epithelial type 2 cells, Alveolar epithelial type 1 cells

## Abstract

**Background:**

Functional alveolar regeneration is essential for the restoration of normal lung homeostasis after acute lung injury (ALI) and acute respiratory distress syndrome (ARDS). Lung is a relatively quiescent organ and a variety of stem cells are recruited to participate in lung repair and regeneration after lung tissue injury. However, there is still no effective method for promoting the proliferation of endogenous lung stem cells to promote repair and regeneration.

**Methods:**

Using protein mass spectrometry analysis, we analyzed the microenvironment after acute lung injury. RNA sequencing and image cytometry were used in the alveolar epithelial type 2 cells (AEC2s) subgroup identification. Then we used Sftpc^+^AEC2 lineage tracking mice and purified AEC2s to further elucidate the molecular mechanism by which CTGF regulates AEC2s proliferation both in vitro and in vivo. Bronchoalveolar lavage fluid (BALF) from thirty ARDS patients who underwent bronchoalveolar lavage was collected for the analysis of the correlation between the expressing of Krt5 in BALF and patients’ prognosis.

**Results:**

Here, we elucidate that AEC2s are the main facultative stem cells of the distal lung after ALI and ARDS. The increase of connective tissue growth factor (CTGF) in the microenvironment after ALI promoted the proliferation of AEC2s subpopulations. Proliferated AEC2s rapidly expanded and differentiated into alveolar epithelial type 1 cells (AEC1s) in the regeneration after ALI. CTGF initiates the phosphorylation of LRP6 by promoting the interaction between Krt5 and LRP6 of AEC2s, thus activating the Wnt signaling pathway, which is the molecular mechanism of CTGF promoting the proliferation of AEC2s subpopulation.

**Conclusions:**

Our study verifies that CTGF promotes the repair and regeneration of alveoli after acute lung injury by promoting the proliferation of AEC2s subpopulation.

**Supplementary Information:**

The online version contains supplementary material available at 10.1186/s12931-023-02512-4.

## Background

The main function of adult stem cells is to maintain tissue homeostasis and to perform repairs after injury [1]. In the lungs, the integrity of the epithelial barrier is essential to prevent pathogen invasion and effective gas exchange. In homeostasis, lung epithelial cells renew slowly but contain region-specific stem cells, which mobilize rapidly to replenish epithelial cells after tissue injury [2]. Functionally defined stem or progenitor cells that have unlimited self-renewal and clonal, pluripotent differentiation in cell hierarchy may be essential for the homeostasis and repair of the lungs [3]. The biology of lung maintenance may be more similar to the organs of endothelial-derived epithelial cells, such as the pancreas and liver. Mature, differentiated or facultative progenitor or stem cells are the main regenerative cells [4].

The distal lung performs essential respiratory functions that can be impaired by inflammation, tumors or infectious diseases. The distal alveolar epithelium is not only crucial for gas exchange but also an important barrier to protect the body from external hazards. For acute injury, alveoli can rapidly repair and regenerate to restore the complete epithelial barrier. The alveolar epithelium contains two main types of epithelial cells: AEC1s and AEC2s. AEC1s are terminally differentiated epithelial cells, and their contribution to alveolar epithelial regeneration is very limited [5]. One of the functions of AEC2s is to release pulmonary surfactant, thus reducing alveolar surface tension to maintain lung morphology during breathing [6]. However, AEC2s are crucial cells in the maintenance of pulmonary homeostasis and regenerate after injury by proliferating and differentiating AEC1s in alveoli [7, 8]. The self-renewal and differentiation of AEC2s must be closely coordinated to maintain tissue integrity and effective repair. This imbalance can lead to severe lung disease [9]. Only a small fraction of mature AEC2s express stem cell function, and their doubling time is approximately 40 days. This stem cell function is induced by alveolar injury and can be activated by dying AEC1s [3, 10]. Recently, with the development of multiomics analysis and single-cell sequencing technology, it has been found that a variety of AEC2 subpopulations participate in the repair and regeneration of lung epithelial cells after acute lung injury and virus infection. A recently identified Axin2^+^ AEC2 subpopulation with constitutive Wnt pathway activity comprises a Wnt signaling niche with fibroblasts near each stem cell. When the alveolar epithelium is severely injured, the Axin2^+^ AEC2 subpopulation proliferates rapidly and participates in the regeneration and repair of the epithelium [11]. Axin2^+^ AEC2s have also been identified in human lungs and are responsible for the growth of AEC2s in alveolar organoids [12]. During alveolar damage, macrophage-derived IL-1β primes a subpopulation of AEC2s expressing Il1r1 via the HIF1a pathway, which is essential for the differentiation of mature AEC1s. Il1r1^+^ AEC2s are another distinct AEC2 lineage population that plays a crucial role in alveolar repair and regeneration [13]. SFTPC^+^ AEC2s may be a heterogeneous population composed of cell subpopulations with different proliferation and differentiation abilities.

After acute lung injury (ALI)/acute respiratory distress syndrome (ARDS), regeneration is crucial for restoring normal lung structure and function. Thus, promoting endogenous regeneration by progenitor or stem cells to restore lung structure and function may be a new therapeutic perspective for the future therapy of pulmonary diseases [9, 14]. This highlight needs to better understand the molecular mechanisms of lung regeneration after lung damage. AEC2s are the main stem / progenitor cells participating in lung repair and regeneration after lipopolysaccharide (LPS) induced ALI [15]. To observe the changes in proteins in the repair microenvironment of AEC2s after ALI, we observed the changes in protein expression in alveoli after acute lung injury by protein mass spectrometry. AEC2s proliferated significantly from 3 days to 5 days post-LPS-induced ALI. During this process, the Hippo-YAP1 signaling pathway was significantly activated, and the pathway inhibitor blocked the proliferation of AEC2s. Therefore, the Hippo-YAP1 pathway plays a crucial role in the proliferation of AEC2s [16]. However, the mechanism is still not well elucidated. The Hippo signaling pathway plays a role in the regulation of cell proliferation, apoptosis and organ size. Mutations or imbalanced regulation of its key proteins will lead to organ dysplasia, multiple cancers, autoimmune diseases and neurodegenerative diseases. Therefore, we speculated that Hippo-YAP1 might be a key signaling pathway for alveolar repair and regeneration after ALI.

Here, we found that the expression of connective tissue growth factor (CTGF), a downstream effector of the Hippo-Yap1 signaling pathway, increased significantly at different time points after acute lung injury. CTGF can promote the proliferation of a subpopulation of AEC2s which expressing Krt5, both in vitro and in vivo. Therefore, we used SFTPC^+^ AEC2 lineage tracking mice to further elucidate the molecular mechanism by which CTGF regulates AEC2s subpopulation proliferation and participates in the repair and regeneration of alveoli after ALI. Our study verifies that CTGF contributes to the regeneration of AEC2s subpopulation to enhance alveolar regeneration and that AEC2s subpopulation are stem cells for alveolar regeneration after acute lung injury.

## Methods

### ALI animal model

Sftpc-CreER^T2^ (Strain #:028054, RRID:IMSR_JAX:028054) and Rosa26-RFP (Strain #:007914, RRID:IMSR_JAX:007914) mouse strains were purchased from the Jackson Laboratory. All mice were maintained in the Daping Hospital animal care facility. We constructed an ALI model according to a protocol described previously [17]. 6 to 8 week-old mice were anesthetized with 60 mg/kg pentobarbital sodium by intraperitoneal injection. Mice were treated with 4.5 mg/kg LPS (Escherichia coli O55:B5, Sigma, L4005) through intratracheal instillation. CTGF (#9237-CT; R&D Systems) treatment was administered via tail vein injection (20 μg/kg) at 24 h after ALI. Mouse experiments were performed on both male and female mice in all conditions, and mice were chosen at random from the cohort but not formally randomized. Animal experiments were carried out under pathogen-free conditions and with randomly chosen littermates of the same sex and matched age and body weight. We conducted all animal care and experimentation in accordance with the Association for Assessment and Accreditation of Laboratory Animal Care guidelines and with approval from the University of Army Medical Animal Care and Use Committees.

### In vivo transfection of AEC2-specific Krt5 knockout virus

The Cre-on adeno-associated Cas9 vector (Cre-on AAV-9) was generated for Krt5 gene knockout. Single guide RNAs (sgRNA-F: CACCGGTACAATGTGGGGGGCTCCAA; sgRNA-R:AAACTTGGAGCCCCCCACATTGTACC) targeting the mouse Krt5 gene were designed and cloned into AAV9-GV725 (flex(CMV)-NLS-SaCas9-NLS-3xHA-bGHpA-U6-sgRNA) by BsaI (Shanghai Genechem Co., Ltd.). After packaging, Cre-on AAV-9 was administered via tail vein injection of Sftpc-CreERT2 and Rosa26-RFP mice from Day 1 (100 μl, 1.5 × 10^11^ v.g./ml). Fourteen days later, the mice were sacrificed, and lung tissue was harvested and used for Krt5 knockdown in AEC2 detection. A negative AAV-9 vector control was also performed.

### QRT-PCR analysis

ReliaPrep RNA Cell Miniprep kit (Promega, # Z6010) was used to isolate RNA from freshly sorted Krt5^+^AEC2s or Krt5^−^AEC2s from alveolar lavage fluid of patients. cDNA was synthesized using Superscript III (Invitrogen, #18,080,085). PCR reaction and analysis was run on Eppendorf Mastercycler ep realplex2. GAPDH was used as internal controls. Forward Sequence for Krt5: GCTGCCTACATGAACAAGGTGG; Reverse Sequence for Krt5: ATGGAGAGGACCACTGAGGTGT.

### FACS analysis and FACS sorting

For FACS sorting, lungs from WT or Sftpc-CreER; tdTomato^+^ mice were collected at 6 to 8 weeks of age and processed into a single-cell suspension using dispase, DNase I and collagenase I, as previously described [15]. The AEC2 population (Sftpc^+^ AEC2 cells) was isolated from the lungs of 6–8-week-old Sftpc-CreER; tdTomato^+^ mice 5 days after induction with 200 μg/g body weight tamoxifen. Krt5^+^ AEC2s and Krt5^−^ AEC2s were sorted by MoFlo SX (Beckman Coulter, Miami FL). Data were analyzed in Summit 5.2 (Beckman). For FACS analysis and sorting of mouse, the following antibodies were used: CD45 (Biolegend, 103,116, 1:100), LysoTracker (Invitrogen, L7526, 1:14000), EpCAM (Biolegend, 118,206, 1:100), Krt5 (Abcam, ab193895, 1:300), and Edu (50 mg/kg, E10187, Life Technologies). For the FACS analysis and sorting of patients, the following antibodies were used: LysoTracker (Invitrogen, #L7526, 1:14000), CD45 (Biolegend, #368,530, 1:1000), KRT5 (ABcam, #ab193895, 1:1000); EpCAM (Biolegend, #324,220, 1:1000).

### Cell culture

AEC2s were cultured in MTEC/Plus, which contained DMEM/F12, 15 mM HEPES, 3.6 mM NaHCO_3_, 4 mM L-glutamine, 10 μg/mL insulin, 5 μg/mL transferrin, 0.1 μg/ml cholera toxin, 5% FBS, 0.01 μm retinoic acid and penicillin/streptomycin [18]. For three-dimensional culture, sorted AEC2s and PDGFRA-GFP^hi^ fibroblasts (1:200) were seeded in a 24-well 0.4-μm Transwell insert (Falcon). Mixed cells were resuspended in MTEC/Plus mixed with 1:1 growth factor–reduced Matrigel (CAT:356,230, BD Biosciences). Then, 500 μL of MTEC/Plus was placed in the lower chamber, and the medium was changed every other day.

### RNA sequencing and bioinformatics analyses

Lineage tracing AEC2s were isolated from mice treated with or without CTGF administration. Total RNA was extracted using a RNeasy Plus Kit (Qiagen, Germany), and 1 μg of RNA was used as input material for the RNA sample preparations. RNA-seq strand-specific libraries were constructed using the VAHTS Total RNA seq (H/M/R) Library Prep Kit (Vazyme, China) according to the manufacturer’s instructions. Cluster was generated by cBot after the library was diluted to 10 pM and then sequenced on the Illumina NovaSeq 6000 platform (Illumina, USA). Library construction and sequencing were performed by Sinotech Genomics Co., Ltd. (Shanghai, China).

We performed a Gene Ontology (GO) analysis for biological processes, cellular components and molecular function and a KEGG pathway analysis (Kyoto Encyclopedia of Genes and Genomes http://www.genome.ad.jp/kegg) via the enrich R package. We classified DEGs according to the official classification of KEGG annotation results and enriched the path function with Phyper. DIAMOND [19] was used to map the DEGs onto the STRING [20] database to determine the interaction between DEG-encoded proteins using homology with known proteins. For the whole interaction result, we provide an input file that can be directly imported into Cytoscape for network analysis.

### Image cytometry

LysoTracker green DND-26 (Invitrogen) was added and incubated with the total lung cell suspension at 37 °C for 30 min. After washing with 2% serum HbSS, the cells were incubated with a mixture of primary antibodies, including anti-CD45 (103,116, Biolegend), anti-EpCAM (118,206, Biolegend) and anti-Krt5 (ab193895, Abcam), in PBS for 30 min on ice. After washing, 20,000 cells of each sample were analyzed by ImageStream®^X^ Mark II. CD45^−^EpCAM^+^LysoTracker^+^Krt5^+^ and CD45^−^EpCAM^+^LysoTracker^+^Krt5^−^ were detected by flow cytometry and image acquisition. Data were analyzed with the IDEAS (Amnis Seattle, USA). Gates for focused and single cells were set according to the manufacturer’s recommendations. All graphs (dot plots, histograms and cell images) and statistics (cell counts and relative amounts) were generated with IDEAS software.

### Quantitative western blotting

Automatic western blots were performed using a Wes automated system (ProteinSimple, California, USA). Purified recombinant proteins were used as calibration standards. Serial dilutions of both the sample and standard were used to determine the linear dynamic range of the assay. According to the ProteinSimple kit (SM-W004), the loading order was: ladder and Experimental protein samples; antibody Diluent II; antibody Diluent II and Primary Antibody; streptavidin-HRP and Secondary HRP Conjugate; luminol-Peroxide mix. SW software was used for data generation and analysis. Antibodies against p-LRP6 (ab226758, 1:50), LRP6 (ab134146, 1:10), p-GSK3b (ab68476, 1:100), GSK3b (ab185141, 1:100), β-catenin (ab16051, 1:50), LEF1 (ab137872, 1:10), and β-actin (ab6276, 1:100) were obtained from Abcam. Antibodies against p-β-catenin (4176, 1:100) were obtained from Cell Signaling Technology.

### RNAscope

The slides were dewaxed, treated with 100% ethanol and then air dried. Then, specimens were processed according to the protocol of the RNAscope Multiplex Fluorescent Reagent Kit v2 (ACD, 323100, USA). After adding the preheated probe, Sftpc (ACD, 314101, USA), Aqp5 (ACD, 430021-C2, USA), Ki67 (ACD, 416771-C3, USA), Krt5 (ACD, 415041-C4, USA), and P63 (ACD, 472561-C3, USA), we pretreated 1 solution for 10 minutes at room temperature and then pretreated 2 for 15 minutes at 99°C. Tissues were pretreated at 40°C for 30 minutes to increase permeability. The preheated Prox1 mRNA probe was incubated with tissue at 40°C for 2 hours. Signal amplification was achieved by continuously applying six amplifiers at 40°C (Amp 1, 2, 3) and room temperature (Amp 4, 5, 6). The signal was detected using a diaminobenzidine mixture on tissue samples for 10 minutes. Then, opal 520, opal 570, opal 690, and opal 620 fluorescent dyes were added to label the probe signal. Nuclear restaining followed the RNAscope procedure and was performed with modified Lille’ hematoxylin (Dako, Carpineria, USA).

### Fluorescence resonance energy transfer detection

Fluorescence resonance energy transfer (FRET) efficiency was assessed by acceptor bleaching. Purified AEC2s were treated with CTGF (10 ng/mL). After droplet formation, imaging was performed on a Leica STELLARIS 5 confocal laser scanning microscope with the LAS X FRET AB module. Briefly, droplets of interest were zoomed in upon, a region in which the Krt5-LRP6 interaction occurred was highlighted and the program was initiated. For photodestruction of the interaction, cells were photobleached with a 488-nm laser line. Images were captured in these channels before and after photobleaching. Approximately 10 droplets were measured in the experiments. The FRET efficiency was calculated as E = (1- Pre/Post) 3100%, where Pre and Post represent the intensity of donor fluorescence before and after photobleaching.

### PCR panel assay of wnt signaling pathway gene expression

The QuantiNova LNA PCR Focus Panel Mouse WNT Signaling Pathway (Qiagen, #249,950 SBMM-043ZA) enables quick gene expression analysis of the WNT signaling pathway using SYBR Green-based qPCR. Total RNA was extracted using a QIAGEN RNeasy Plus Kit. First-strand cDNA was synthesized from 1 μg of RNA using the iScript cDNA Synthesis kit (#1,708,890, Bio–Rad). Briefly, 20-μl reactions were prepared by combining 4 μl of iScript Select reaction mix, 2 μl of gene-specific enhancer solution, 1 μl of reverse transcriptase, 1 μl of gene-specific assay pool (20×, 2 μM), and 12 μl of RNA diluted in RNase-free water. Quantitative real-time PCR was carried out using synthesized cDNA, primers, and SsoFast EvaGreen Supermix (#172–5204, Bio–Rad). The expression levels of target genes were calculated using the ddCt method relative to the expression of a housekeeping gene, β-actin. The data shown are the relative quantity (RQ), with the RQ of the control cells set to one.

### Histology and immunostaining

Lungs were fixed with 4% paraformaldehyde in phosphate-buffered saline. Immunofluorescence staining was performed on cryosections or paraffin sections. Primary antibodies and dilutions were as follows: Sftpc (Millipore, Ab3786, 1:1,000), Aquaporin 5, AQP5 (Abcam, ab78486, 1:1,000), T1a (Sigma, P995, 1:400), Edu (Beyotime, C0081S), Ki67 (Abcam, ab231172, 1:400), Krt5 (Abcam, ab193895, 1:300), EpCAM (Abcam, ab221552, 1:200), CD45 (Biolegend, 103,116, 1:100), Lysotracker (Invitrogen, L7526), and p63 (Abcam, ab735, 1:200). The secondary antibodies were Alexa Fluor 488 (Abcam, ab150081, 1:400) and Alexa Fluor 594 (Abcam, ab150120, 1:400). Images were obtained using a Zeiss confocal microscope and Delta Vision Elite (Applied Precision).

### Patient samples

The BALF samples of 30 ARDS patients were collected from Daping Hospital of the Army Medical University. The collection of samples was approved by the institutional review board. Basic demographic and clinical data, including age, sex, partial pressure of arterial oxygen/fraction of inspired oxygen (PaO_2_/FiO_2_), timing of the diagnosis of ARDS and clinical outcomes, were retrieved from the registry (Table S1). Patients were excluded if they had preexisting respiratory, cardiovascular, renal, hepatic, immunologic or hematologic diseases. Thirty ARDS patients were stratified into mild, moderate, and severe ARDS according to chest imaging, origin of edema and PaO_2_/FiO_2_[21].

### Data analysis and statistics

Unpaired Student’s t tests were used to compare the means of two groups. One-way analysis of variance (ANOVA) was used for comparisons among several groups. When ANOVA yielded significant differences, post hoc testing of differences between groups was performed using the least significant difference (LSD) test. The Kaplan–Meier method was used to comparedifferences in mortality rates between groups. Statistical significance was set at P values < 0.05. GraphPad Prism 7.0 software was used for statistical analysis, and statistical charts were generated. Adobe Illustrator CC (version 18.0.0, 32-bit) and Adobe Photoshop CS6 software (version 13.0.1, 64-bit) were used to construct mechanism diagrams and reasonably adjusted pictures.

## Results

### CTGF is an effective cytokine accelerating alveolar repair by promoting the proliferation of AEC2s after ALI

**Fig. 1:**
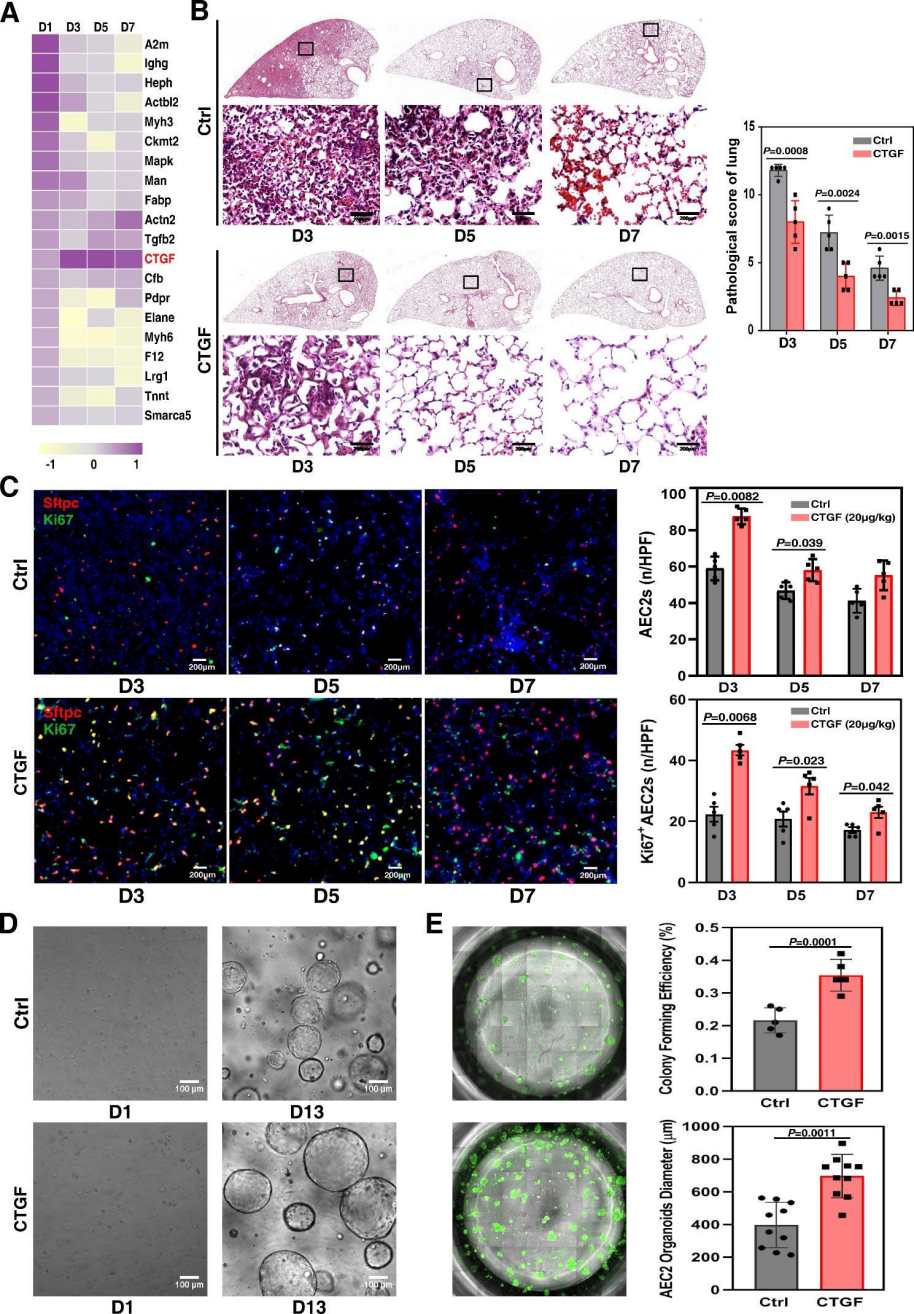
CTGF promotes the proliferation of AEC2s post ALI. (**A**) Heatmap of differentially expressed proteins in mouse lungs from 1 to 7 dpi (D1 to D7). (**B**) Representative histological sections of sham control and CTGF-treated mouse lungs from 3 to 7 dpi. H&E staining. Scale bar, 200 μm. (**C**) Immunofluorescence (IF) staining of Sftpc and Ki67 in control (ctrl) and CTGF-treated mouse lungs from 3 to 7 dpi (*P* = 0.0082; *P* = 0.039; *P* = 0.0068; *P* = 0.023; *P* = 0.042 by two-tailed *t* test). All bar graphs show the mean ± SEM of three independent experiments. n / HPF, number / high power field. Scale bar, 200 μm. (**D**) Representative differential interference contrast images of control and 10 ng/ml CTGF-treated AEC2 organoids from Day 1 to Day 13. Scale bar, 100 μm. (**E**). Representative fluorescence microscopy images of control and CTGF-treated AEC2 organoids at Day 13. Calcein-AM staining was used to stain AEC2 organoids, and organoid size and colony-forming efficiency at Day 13 were analyzed. All bar graphs show the mean ± SEM of three independent experiments. *P* = 0.001 and *P* = 0.0011 by two-tailed *t* test. Scale bar, 50 μm

The results of protein mass spectrometry showed that the expression of CTGF in the lung tissue microenvironment was evidently elevated from 3 days to 7 days post-LPS-induced ALI (Fig. 1A and Data file S1) [22, 23]. Then, we observed the effect of CTGF on alveolar repair in an ALI mouse model. Histological analysis indicated that 20 μg/kg CTGF-treated lung tissue displayed better alleviated alveolar tissue damage at 3 dpi compared with the control group. At 7 days post-LPS-induced ALI (7 dpi), the lung alveolar morphology of the CTGF-treated group had recovered, while that of the control group generally recovered at 14 days (Fig. 1B). We used lineage-labeled mouse AEC2s and Sftpc-CreER^T2^; Rosa26‐RFP double heterozygous mice (Sftpc‐CreER^T2^; Rosa26‐RFP) to observe the effect of CTGF on the proliferation of AEC2s post ALI. The results showed that both the number of AEC2s and proliferated AEC2s in the CTGF-treated group were significantly increased from 3 to 7 dpi (Fig. 1C). We further observed the effect of CTGF on the proliferation of AEC2 organoids in vitro. Flow cytometry-sorted AEC2s were cultured in Matrigel to form AEC2 organoids. CTGF-treated AEC2 organoids revealed significant increases in organoid size and colony-forming efficiency (CFE) compared with the control group at 13 days (Fig. 1D and E). As indicated by RNAscope, the transcriptional level of Ki67 was highly expressed in CTGF-treated AEC2 organoids. The protein expression levels of the AEC2-specific protein Sftpc were also significantly increased in the CTGF-treated group (Fig. S1). These results indicated that CTGF-treated AEC2s were more proliferative.

To prevent pulmonary fibrosis caused by a high dose of CTGF, we observed the relationship between the CTGF concentration gradient and pulmonary fibrosis. Three doses of CTGF (20, 100 and 200 μg/kg) were used to treat LPS-induced ALI mice. After 7 days of continuous administration of 20 μg/kg CTGF, Masson staining showed that there was no fiber staining in the lung. However, 100 and 200 μg/kg CTGF caused fiber deposition in the alveolar septum, indicating that more than 100 μg/kg CTGF treatment will cause pulmonary fibrosis (Fig. S2A-2 C). Therefore, 20 μg/kg is a safe dose and will not cause pulmonary fibrosis. Taken together, these results indicate that CTGF can promote the proliferation of AEC2 organoids after ALI.

### Proliferated AEC2s mediate alveolar repair via differentiation to AEC1s

**Fig. 2:**
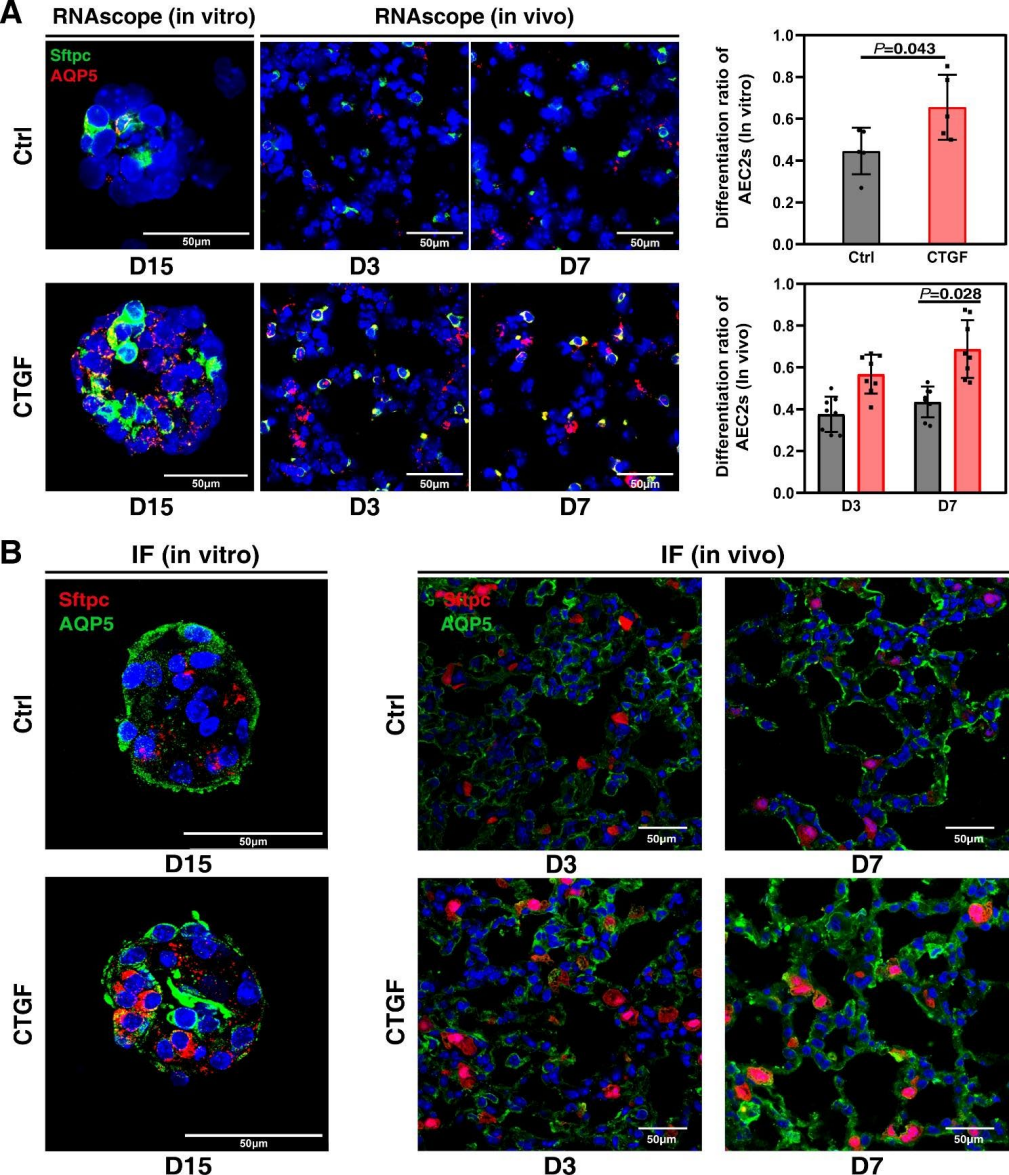
AEC2s mediate alveolar repair via differentiation to AEC1s. (**A**) High-magnification images of RNAscope staining of Sftpc and AQP5 in AEC2 organoids (left) and lung tissue sections (right). All bar graphs show the mean ± SEM of three independent experiments. *P* = 0.043; *P* = 0.028 by two-tailed *t* test. Scale bar, 50 μm. (**B**) High-magnification images of the immunofluorescence staining of Sftpc and AQP5 of the AEC2 organoids (left) and lung tissue sections (right). Scale bar, 50 μm

AEC2s proliferate and differentiate into AEC1s to participate in the repair and regeneration of alveoli injury after ALI/ARDS [1]. Therefore, we observed the differentiation of proliferating AEC2s to further clarify the role of CTGF treatment in the repair of ALI. Using clonal alveolar organoid culture and CTGF treatment in vitro, AEC2s formed a greater number of and larger organoids that contained more AEC1s and AEC2s than the control group, as shown by RNAscope at the transcriptional level. The numbers of AEC1 cells in AEC2 organoids were greater than those in the control group, indicating that CTGF treatment elevated the differentiation ratio of AEC2s in vitro (Fig. 2A). The proliferation of AEC2s treated with CTGF for 3 days was significantly accelerated compared with that of the control group. Consistent with the transcriptional level, the translational levels of AQP5 were higher than those of the control group after 7 days of CTGF treatment, indicating that the differentiation of AEC2s was significantly increased (Fig. 2B). Taken together, these results demonstrate that CTGF promotes the differentiation of AEC2s into AEC1s, thus promoting the repair and regeneration of alveoli after ALI.

### CTGF treatment induces the proliferation of AEC2s subpopulation post ALI

**Fig. 3:**
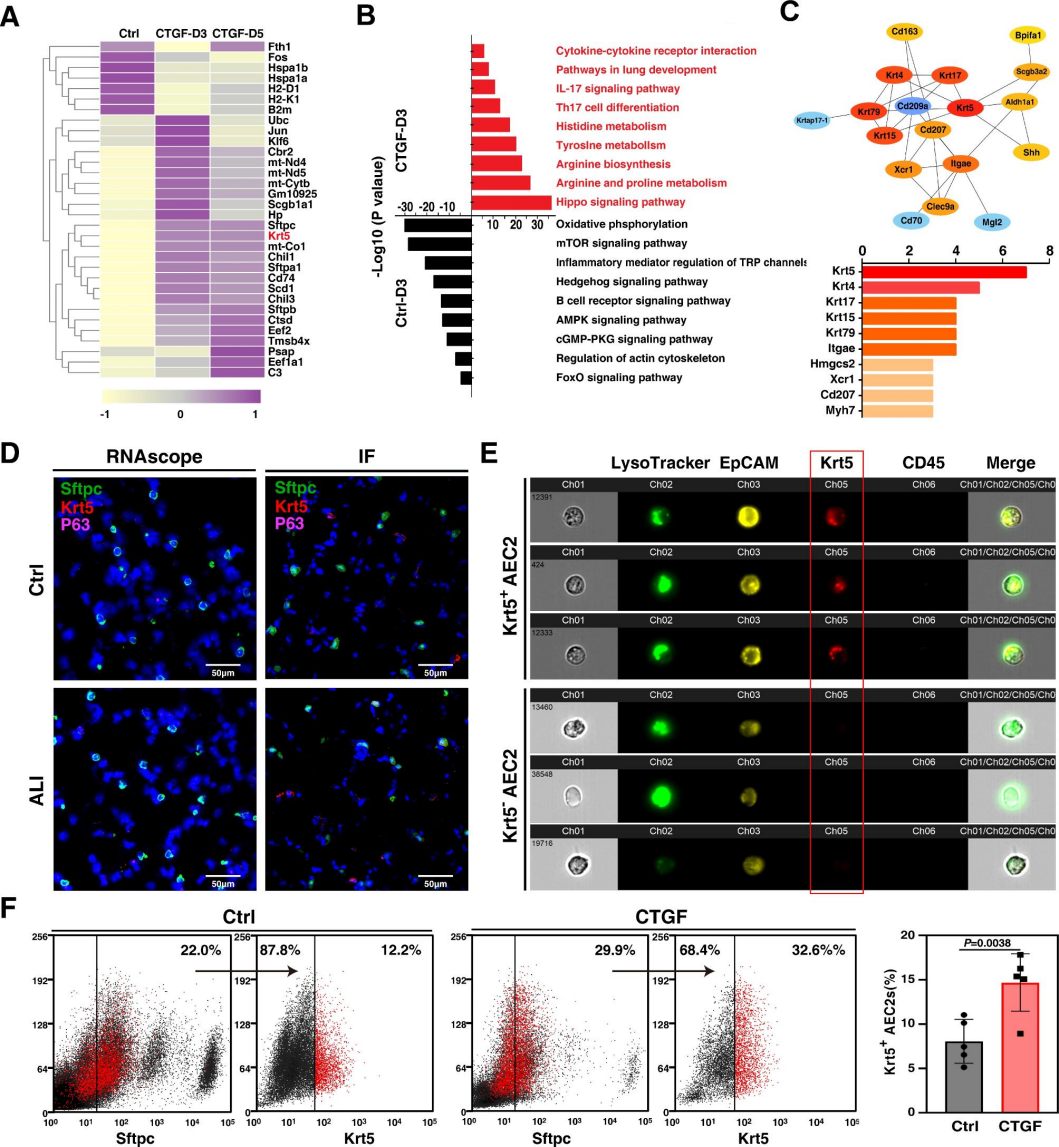
CTGF treatment increased the number of AEC2 subpopulations in the mouse lung. (**A**). Heatmap of the top 30 upregulated genes in CTGF-treated mouse AEC2s compared with nontreated controls. (**B**). Enriched Gene Ontology classes of AEC2s isolated from CTGF-treated 3 dpi ALI mouse lung tissue compared with control. Red bar, GO class of upregulated genes in AEC2s isolated from CTGF-treated 3 dpi ALI mouse lungs. Black bar, GO class of upregulated genes in AEC2s isolated from sham control mouse lungs. GO terms were ranked by the enrichment *P* value. (**C**) STRING analysis of the protein–protein interaction network of highly expressed genes in AEC2s isolated from CTGF-treated 3 dpi ALI mouse lungs compared with the control. (**D**) The mRNA and protein expression levels of Sftpc, Krt5 and p63. Scale bar, 50 μm. (**E**) Krt5 expressing AEC2s subpopulations were detected by image cytometry. LysoTracker-pos EpCAM-pos was used as an AEC2 marker protein, and CD45-negative cells could exclude cells from the blood system. Images are representative of 3 independent experiments. (**F**) Flow cytometry was used to observe the effect of CTGF treatment on the proportion of Krt5 expressing AEC2s in mouse lungs (n = 5; 8.30% ± 2.47% in the control group vs. 14.98% ± 3.72% in the CTGF-treated group, *P* = 0.0038 by two-tailed *t* test)

To observe the effect of CTGF on the transcriptomic profiles of AEC2s post ALI, we performed RNA sequencing (RNA-seq) analysis of AEC2s isolated from ALI mouse lungs after treatment with CTGF. We identified the differentially expressed genes in AEC2s isolated from CTGF-treated 3 dpi and 5 dpi mouse lungs compared with the control group. The transcription levels of some genes were significantly elevated, including Sftpc, Krt5, mt-Co1, Chil1, Sftpa1, Cd74, Scd1, Chil3, and Sftpb. (Fig. 3A and Data file S2). Further analysis of gene ontology revealed that CTGF treatment enhanced the activation of pathways in lung development, cytokine–cytokine receptor interaction, metabolism of multiple amino acids and the Hippo-YAP1 proliferation signaling pathway. In AEC2s isolated from nontreated ALI mouse lung tissue at 3 dpi, oxidative phosphorylation, the mTOR signaling pathway, the B cell receptor signaling pathway, the AMPK signaling pathway, the FOXO signaling pathway and other inflammation-related pathways were upregulated (Fig. 3B). These data suggested that CTGF might promote alveolar repair by regulating metabolism-, inflammation- and development-related pathways of AEC2. For AEC2s isolated from CTGF-treated 3 dpi ALI mouse lungs compared with control mice, protein–protein interaction network analysis of over-expressed genes identified that Krt5 interacted with most kinds of proteins (krt17, krt15, krt4, Shh, ALDH1A1, cd209a and scgb3a2) (Fig. 3C). CTGF treatment altered the transcriptomic profiles of AEC2s post ALI. The transcription level of Krt5 was elevated significantly in AEC2s post ALI from 3 dpi to 5 dpi.

Recent studies have shown that p63^+^Krt5^+^ distal airway stem cells (DASCs) undergo proliferative expansion in response to influenza-induced lung injury and assemble into nascent alveoli at sites of interstitial lung inflammation. p63^+^Krt5^+^ DASCs play pivotal roles in the repair and regeneration of alveoli after injury [24, 25]. Therefore, we observed the expression of Krt5 and p63 in the alveoli of mouse lungs after ALI. We detected the mRNA transcription and protein expression of Krt5 and p63 in AEC2s (Fig. 3D). Significantly, no p63 and Krt5 dual-positive AEC2s appeared in the alveolar area, and only Krt5 expressing was detected in AEC2s after CTGF treatment post ALI. Then, we validated the Krt5 expressing AEC2s subpopulation by imaging flow cytometry (IFCM). LysoTracker and EpCAM were used to identify AEC2s [26]. Two subpopulations of AEC2s, CD45^−^ LysoTracker^+^ EpCAM^+^ Krt5^+^ and CD45^−^ LysoTracker^+^ EpCAM^+^ Krt5^−^, could be found in mouse lungs (Fig. 3E). The results of flow cytometry also showed that the proportion of Krt5 expressing AEC2s in ALI mice increased after CTGF treatment (Fig. 3F), indicating that CTGF treatment increased the proliferation of AEC2s subpopulation in mouse lungs after ALI.

### Krt5 expressing AEC2s are a crucial subpopulation of stem cells in lung regeneration

**Fig. 4:**
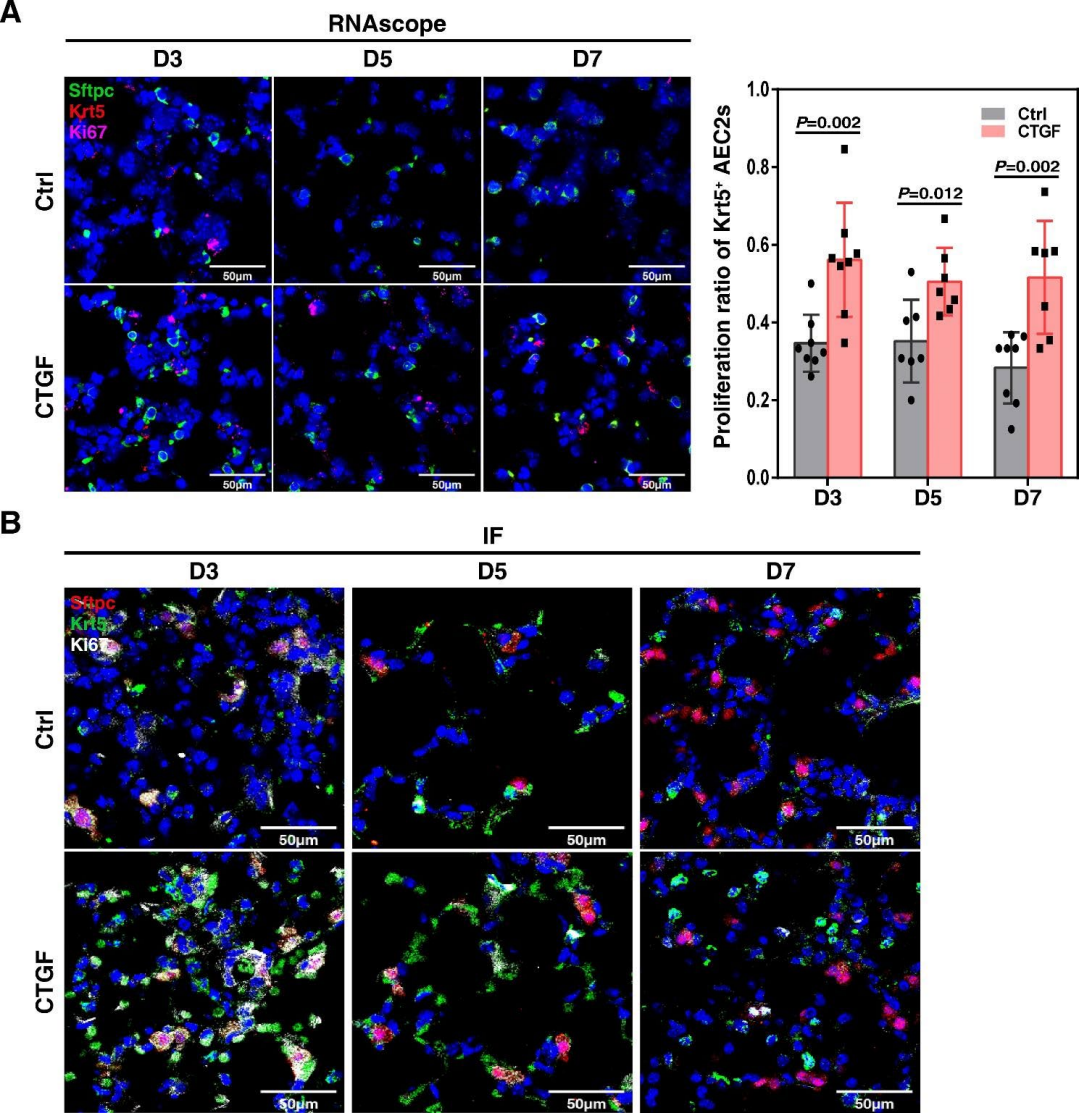
Krt5 expressing AEC2s are an important progenitor subpopulation of lung regeneration after ALI. (**A**) The proliferative potential of Krt5 expressing AEC2s post ALI observed by RNAscope. Scale bar, 50 μm. (B) The proliferative potential of Krt5 expressing AEC2s post ALI observed by immunofluorescence staining. Scale bar, 50 μm

Then, Krt5 expressing AEC2s’ self-renewal capability was observed by Ki67 staining with or without CTGF treatment post ALI at both the transcriptional and translational levels. In damaged areas, clonal proliferation of Ki67^+^ Krt5 expressing AEC2s was observed in the alveolar area at 3 dpi in the CTGF-treated group. The proliferation of Ki67^+^ Krt5 expressing AEC2s gradually returned to normal at 5 to 7 dpi (Fig. 4A and B). Quantitative analysis showed that the proportion of proliferated Krt5 expressing AEC2s in the CTGF treated group was significantly higher than that in the control group at 3–7 dpi (*P* = 0.002; *P* = 0.012; *P* = 0.002 by two-tailed *t* test). RNA-seq analysis of Krt5^+^ AEC2s and Krt5^−^ AEC2s suggested that Krt5^+^ AEC2s possess a distinct gene expression profile enriched in cell proliferation, epithelial development and lung morphogenesis genes (Fig. S3). In addition, we compared Krt5^+^ AEC2s and Axin2^+^ AEC2s. As shown in Fig. S4 that most AEC2s did not co-express Krt5 and Axin2. Only a few AEC2s cells co-express Axin2 and Krt5. After CTGF treatment, the number of Krt5^+^ AEC2s and Axin2^+^ AEC2s all increased significantly (Fig. S4). Taken together, the proportion of proliferated Krt5 expressing AEC2s increased after CTGF administration, and Krt5 expressing AEC2s had higher proliferation potential than other AEC2s, indicating that CTGF treatment promoted the increase in Krt5 expressing AEC2s in mouse lungs after ALI and that Krt5 expressing AEC2s are an important subpopulation of stems/progenitors in lung regeneration after ALI.

### AEC2-specific knockout of Krt5 confirmed the essential role of Krt5 in lung repair and regeneration post ALI

**Fig. 5:**
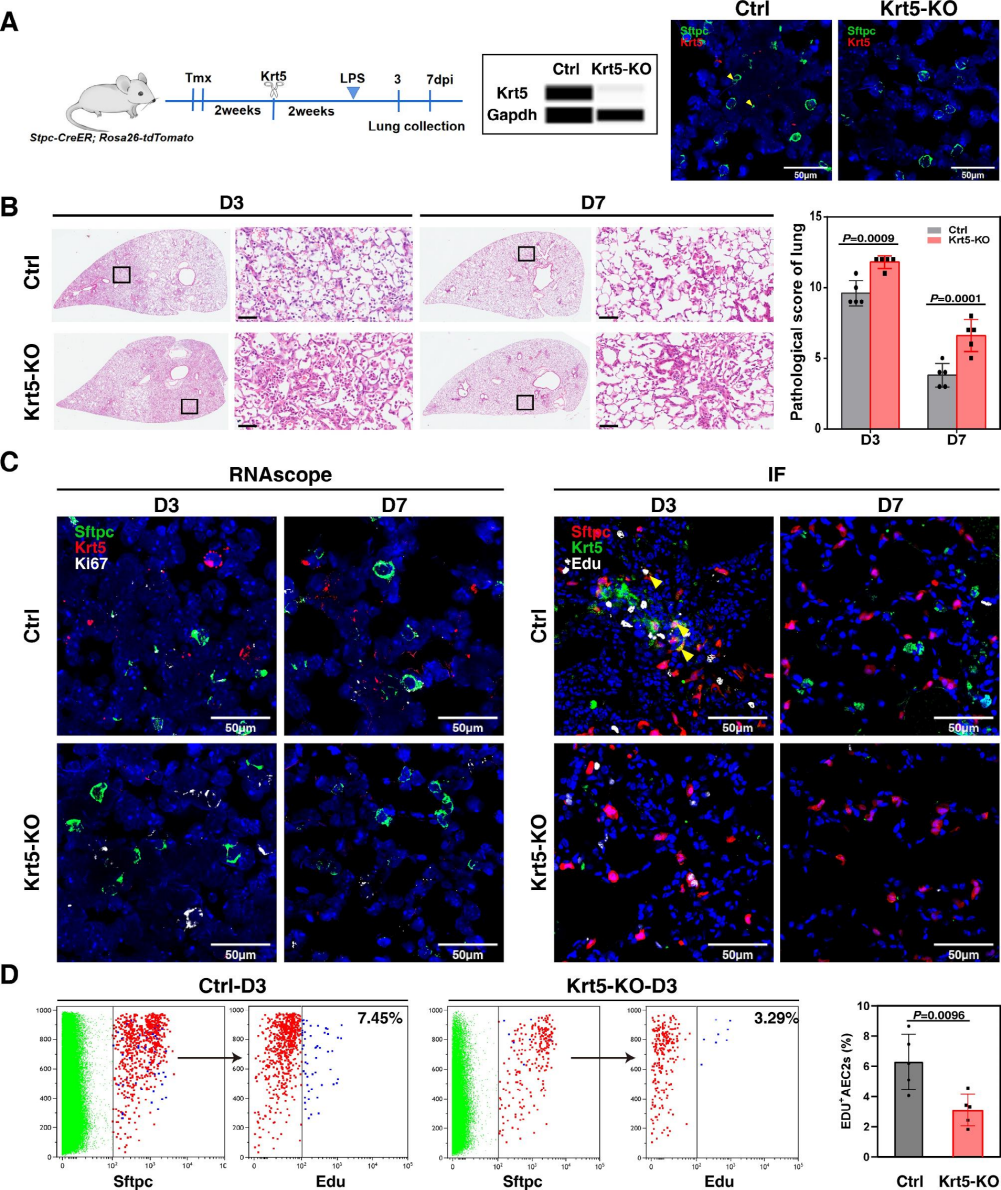
AEC2-specific knockout of Krt5 confirmed the essential role of Krt5 in lung repair and regeneration post ALI. (**A**) Sftpc-CreER^T2^ and Rosa26-RFP mice were injected with the Krt5 Cre-on AAV-9 vector two weeks after tamoxifen administration. The transcriptional and translational levels of Krt5 in AEC2s were verified by quantitative Western blot and RNAscope analyses. (**B**) Representative histological sections of control and Krt5 knockout mouse lungs from 3 to 7 dpi (*P* = 0.0009 for 3 dpi ; *P* = 0.0001 for 7 dpi by two-tailed *t* test). H&E staining. Scale bar, 200 μm. (**C**) High-magnification images of RNAscope staining of control and Krt5 knockout lung tissue sections at 3 and 7 dpi (left) and immunofluorescence (right). Scale bar, 50 μm. (**D**) Flow cytometry analysis to detect the proportions of proliferating AEC2s post ALI in the control and Krt5 knockout groups (n = 5; 6.48% ± 1.46% for the control group; 3.27% ± 1.12% for the Krt5 knockout group, *P* = 0.0096 by two-tailed *t* test)

To verify the role of Krt5 in lung repair and regeneration post ALI, a Cre-on adeno-associated Cas9 vector (Cre-on AAV-9) was generated for Krt5-specific knockout in mouse AEC2s. Sftpc-CreER^T2^ and Rosa26-RFP mice were injected with the vector, and Krt5 expression was specifically knocked out in AEC2s, which was confirmed by both Western blot and RNAscope analysis (Fig. 5A). A large number of dead epithelial cells and exudative inflammatory cells were found in the alveoli in the Krt5 knockout group, and the alveolar structure was not recovered at 7 dpi. However, the non-Krt5 knockout group recovered at 7 dpi, and the alveolar morphology returned to normal (Fig. 5B). Then, the effect of Krt5 knockout on AEC2 self-renewal ability was observed at the transcriptional and translational levels by proliferation staining post ALI in the Krt5 knockout group and the control group. In damaged areas, proliferation of Krt5 expressing AEC2s and all AEC2s were observed in the alveolar area at 3 dpi in the control group. The proliferation of Krt5 expressing AEC2s in all AEC2s gradually returned to normal at 7 dpi. In the Krt5 knockout group, Ki67^+^ AEC2s were rare (Fig. 5C). Flow cytometry analysis showed that the proportion of AEC2 proliferation post ALI in the Krt5 knockout group was significantly lower than that in the non-Krt5 knockout group (control group) (6.48% ± 1.46% for the control group and 3.27% ± 1.12% for the Krt5 knockout group, *P* = 0.0096) (Fig. 5D). These results verified that Krt5 expressing AEC2s are critical in alveolar epithelial repair and regeneration through a direct effect on AEC2 proliferation.

### Krt5 promotes the expansion of Krt5 expressing AEC2s by binding to LRP6 and activating the Wnt signaling pathway

**Fig. 6:**
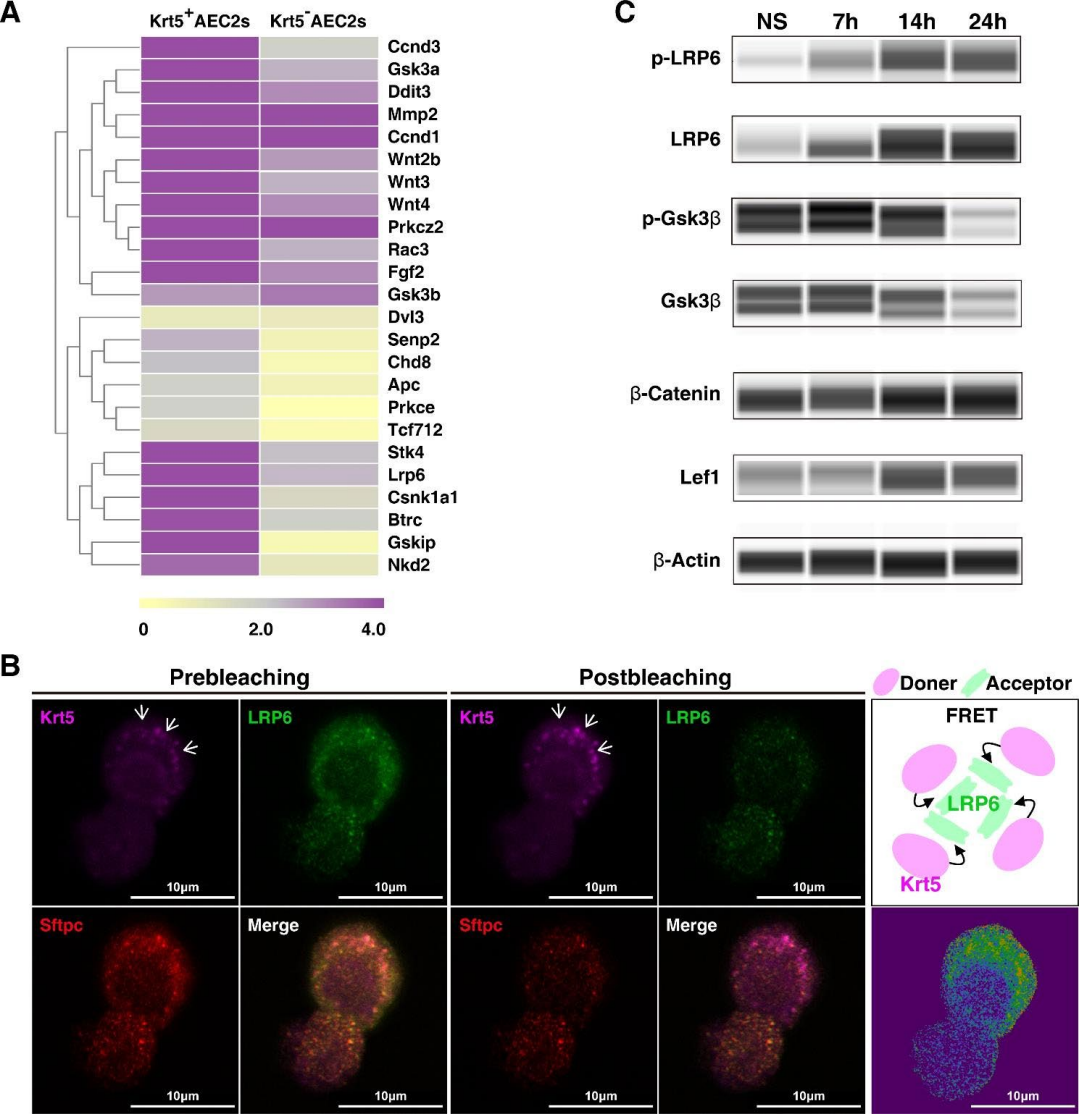
Krt5 promotes the expansion of Krt5 expressing AEC2s by binding to LRP6 and activating the Wnt signaling pathway. (**A**) Heatmap of the expression levels of key genes in the Wnt signaling pathway in Krt5 expressing AEC2s and Krt5^−^ AEC2s by PCR panel assay. (**B**) Purified AEC2s were treated with CTGF (10 ng/mL), and confocal FRET analysis was used to verify the Krt5 and LRP6 interaction. Images of the donor (Krt5) and acceptor (LRP6) before and after acceptor photobleaching show a strong recovery of donor fluorescence in the bleached area (marked by white arrow), indicating an interaction between Krt5 and LRP6 on AEC2s. Scale bar, 10 μm. (**C**) Western blot analysis of the indicated protein expression in Krt5 expressing AEC2s after CTGF (10 ng/ml) stimulation for 7 to 24 h

Given that Krt5 exacerbated ALI and postponed lung repair and regeneration after Krt5-specific knockout in AEC2s, we concluded that Krt5 is a functional molecule participating in repair and regeneration post ALI. We further explored the mechanism. CTGF is a key cytokine that mediates the proliferation of Krt5 expressing AEC2s, and its receptor is LDL receptor-related protein 6 (LRP6), which is also the receptor of the Wnt signaling pathway. The Wnt signaling pathway has previously been shown to have important roles in the expansion of alveolar stem/progenitor cells and in lung repair and regeneration [12, 27]. Therefore, we speculate that CTGF may regulate the proliferation of Krt5 expressing AEC2s through LRP6-Wnt signaling. Then, we verified the expression of the main genes in the Wnt signaling pathway in vivo by PCR panel assay. The expression levels of the main genes in the Wnt signaling pathway, such as Ccnd3, Gsk3a, Ddit3, Wnt3, Wnt4, and Lrp6, were significantly increased in Krt5 expressing AEC2s compared with Krt5^−^ AEC2s isolated from ALI mouse lungs at 3 dpi. (Fig. 6A). When mediated by CTGF, there may be an interaction between Krt5 and LRP6 in AEC2s. Therefore, we performed fluorescence resonance energy transfer (FRET) assays to observe the protein interaction between Krt5 and LRP6. Purified AEC2s were treated with CTGF, and confocal FRET analysis verified the interaction between Krt5 and LRP6 on AEC2s (Fig. 6B). A hallmark of the Wnt pathway is that via the inhibition of glycogen synthase kinase 3 beta (GSK-3β), β-catenin protein accumulates in the cytoplasm and subsequently translocates to the nucleus, where it engages in gene transcription [28, 29]. To observe whether the binding of Krt5 and LRP6 mediates the activation of Wnt signaling and promotes the amplification of Krt5 expressing AEC2s, we analyzed the phosphorylation and activation of LRP6, GSK3β, β-catenin and LEF1 in Krt5 expressing AEC2s by an automated immunoblot method. The results showed that the phosphorylation of membrane LRP6 was significantly increased at 7 h and reached a peak at 14 h after stimulation with LPS and CTGF. GSK3b in the cytoplasm was significantly phosphorylated at 7 h, and β-catenin in the nucleus accumulated the most at 24 h (Fig. 6C). Taken together, these results show that Krt5 promotes the phosphorylation of the Wnt signaling coreceptor LRP6 by binding with it and then activates the Wnt signaling pathway of Krt5 expressing AEC2s, which is the mechanism by which Krt5 expressing AEC2s participate in the repair and regeneration of alveoli after ALI.

### The expression of Krt5 in BALF is correlated to the severity of ARDS

**Fig. 7:**
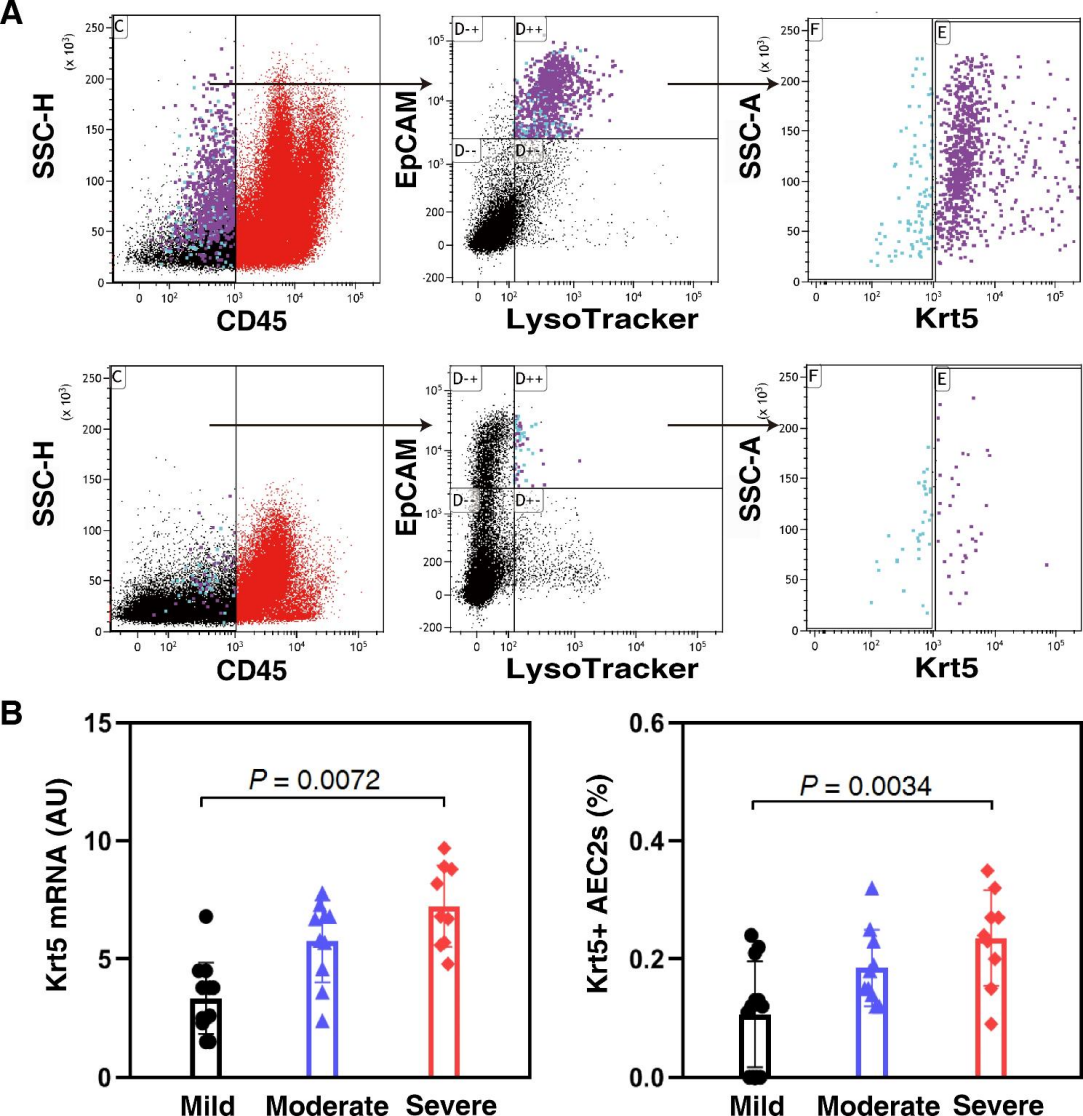
Association between the number of Krt5 expressing AEC2 subpopulations and the severity of ARDS. (**A**) Representative flow cytometry analysis of Krt5 expressing AEC2 subpopulation percentages in the BALF of control and ARDS patients. (**B**) Quantitative analysis of Krt5 mRNA and Krt5 expressing AEC2 subpopulation percentages in the BALF of mild, moderate and severe ARDS patients (*P* = 0.0072 and *P* = 0.0034 by one-way ANOVA).

We next investigated the clinical relevance of the Krt5 expression in bronchoalveolar lavage fluid (BALF) to the severity of ARDS in patients. According to the Berlin Definition [21], our study enrolled 30 patients who met the criteria of ARDS. PaO_2_/FiO_2_ thresholds were used to stratify ARDS severity (mild, moderate, severe), among which mild (n = 12, 40.0%), moderate (n = 10, 33.3%) and severe (n = 8, 26.7%). The demographic and clinical characteristics of the patients are shown in Table S1. We observed the proportions of exfoliated Krt5 expressing AEC2s in BALF of these patients by CD45, lysoTracker, EpCAM and Krt5. The results showed that there was a CD45^−^ Krt5^+^ lysoTracker^+^ EpCAM^+^ AEC2 subpopulation in human BALF (Fig. 7A). BALF Krt5 mRNA levels were significantly correlated with the severity of ARDS. The mRNA expression level of BALF Krt5 in the severe group was higher than those in the moderate and mild groups (*P* < 0.01 compared with severe and mild ARDS patients) (Fig. 7B). Moreover, there was also a correlation between the severity of ARDS and the proportion of exfoliated Krt5 expressing AEC2s in BALF. The proportion of exfoliated Krt5 expressing AEC2s in the severe group was higher than those in the moderate and mild groups (*P* < 0.01 compared with severe and mild ARDS patients) (Fig. 7B). These findings further support a crucial role for Krt5 expressing AEC2s in human acute lung injury and may be a potential biomarker of ARDS severity.

## Discussion

The pathological basis of ALI/ARDS is diffuse alveolar injury. The severity of alveolar injury directly affects gas exchange function and determines the prognosis of patients with ALI/ARDS. Therefore, ameliorating alveolar tissue repair and regeneration after ALI and restoring the normal structure and function of the blood-air barrier is an effective strategy to treat ALI/ARDS. The lung is an organ with certain self-repairing potential, and injury is an essential factor to activate the self-repairing potential of lung tissue [30]. Systematically revealing the self-repair mechanism and regulation of alveolar epithelial cells after ALI may provide prevention and treatment measures for ALI/ARDS.

Therefore, many studies have focused on exploring the stem/progenitor cell populations involved in alveolar repair and regeneration. Zuo et al. elucidated a group of distal airway basal stem cells of Krt5^+^p63^+^ (distal airway stem cells, DASCs), which give rise to collections of Krt5^+^ basal cells (or pods) in severely damaged areas of H1N1 influenza-injured mouse lungs and then proliferate and differentiate and participate in alveolar repair and regeneration [25, 31, 32]. In this study, we found that there were almost no Krt5 positive cells in the alveoli when the lung was in homeostasis. However, after ALI induced alveolar damage, a group of Krt5^+^SPC^+^ cells appeared in the alveoli. They did not express p63 at either the transcriptional or protein level. One of the limitations of our study is that we did not use the Krt5 lineage mice to track the origin of this subgroup of Krt5 and SPC positive cells in vivo. Therefore, it is unclear whether this subgroup of cells originated from the airways. They might also be p63^+^Krt5^+^SPC^−^ airway cells that migrated to the alveoli and might be mobilized to acquire SPC expression. Then these cells are stimulated by residual tamoxifen in mice to obtain SPC lineage markers. So they were not AEC2s in the beginning, but lose p63 expression after acute lung injury and acquire SPC. It is also possible that p63^−^Krt5^+^airway cells might migrate to the alveoli and express SPC.

Effective and coordinated tissue repair is crucial to maintain the integrity and function of the lung. When responding to inflammatory assault, it is critical to perceive the changes in the inflammatory environment and start repair and solve the damage according to the corresponding inflammatory factor microenvironment. Here, we elucidated some new findings related to endogenous stem/progenitor cells in lung repair after acute lung injury. First, we provide evidence by protein mass spectrometry analysis that CTGF treatment can significantly promote the repair and regeneration of alveoli after ALI and improve the survival rate of mice post ALI. Organoid analysis showed that CTGF can strongly promote the proliferation of AEC2s in vivo. CTGF is a double-edged sword for lung development and the maintenance of homeostasis. It plays an important role in the proliferation and differentiation of AEC2s. However, when excessive, it may cause pulmonary fibrosis [33–35]. We observed a dose-dependent effect between CTGF and pulmonary fibrosis after ALI. Twenty μg/kg is a safe dose and can promote alveolar repair after ALI without causing pulmonary fibrosis. Doses greater than 100 μg/kg can cause pulmonary fibrosis.

Then, we found that a group of SPC^+^ cells also expressed Krt5 through RNA-seq analysis of AEC2 treated with CTGF after ALI. Image flow cytometry confirmed the expression of Krt5 in SPC^+^ subpopulation and found that they are important stem / progenitor cells involved in mouse alveolar repair and regeneration after ALI. The proportion of proliferated Krt5^+^SPC^+^ cells in AEC2s increased after CTGF administration, and they had stronger proliferation potential than Krt5^−^SPC^+^ cells, indicating that CTGF administration promoted the increase in Krt5 expressing AEC2s in mouse lungs after ALI and that Krt5^+^SPC^+^ cells are an important subpopulation of stems / progenitors in lung regeneration after ALI. LRP6 is a co-receptor with the CTGF and Wnt signaling pathways. Wnt signaling is a key pathway in modulating the AEC2-to-AEC1 transition and regulates the proliferation of AEC2s [12, 36]. CTGF can regulate the proliferation of Krt5^+^SPC^+^ cells by activating LRP6 phosphorylation and the Wnt signaling pathway can then promote the repair and regeneration of alveoli. Krt5^+^SPC^+^ cells are a crucial subpopulation that promote the regeneration of functional alveolar epithelium by regeneration of AEC2s after acute lung injury. We also confirmed the presence of Krt5^+^SPC^+^ cells in human bronchoalveolar lavage fluid and human distal lung tissue. The preservation and accessibility of mouse and human Krt5^+^SPC^+^ cells provide an opportunity to elucidate the mechanism of human lung stem/progenitor cell biology and contribute to the development of new therapies for acute lung injury. The proportion of Krt5^+^SPC^+^ cells in severe ARDS patients was higher than those in moderate and mild ARDS patients, further supporting an essential role of Krt5^+^SPC^+^ cells in human acute lung injury and suggesting that Krt5^+^SPC^+^ cells may be a potential biomarker for ARDS severity. In summary, our study verifies that CTGF promotes the repair and regeneration of alveoli after acute lung injury by promoting the proliferation of Krt5^+^SPC^+^ cells which are stem cells in alveolar repair and regeneration.

## Conclusions

Our study elucidated that CTGF promotes the repair and regeneration of alveoli after acute lung injury by promoting the proliferation of Krt5 expressing AEC2s in a LRP6-Wnt signaling pathway-dependent manner, which may shed light on the mechanism of lung tissue repair after ALI and provide a potential therapeutic target for ALI and ARDS.

### Electronic supplementary material

Below is the link to the electronic supplementary material.

**Additional file 1: Fig. S1**. CTGF enhance the growth of AEC2s in vitro. (A) High-magnification images of the RNAscope staining of Sftpc, AQP5 and Ki67 of the AEC2s organoids. Scale bar, 50 μm. (B) High-magnification images of the immunofluorescence staining of Sftpc and T1a of the AEC2s organoids. Scale bar, 50 μm

**Additional file 2: Fig. S2**. Dose effect of CTGF and mouse pulmonary fibrosis. (A) Masson staining was observed in lung tissue after 7 days of continuous administration of 20 ug/kg CTGF, no obvious fiber staining was found in lung tissue. (B) After continuous administration of 100 ug/kg CTGF for 7 days, there was a small amount of fiber deposition in the alveolar septum (black arrow indicates fiber deposition). (C) Significant pulmonary fibrosis was observed after 7 days of continuous administration of 200ug/kg CTGF. Scale bar, 200 μm

**Additional file 3: Fig. S3**. RNA-seq analysis of Krt5 expressing AEC2s and Krt5^−^ AEC2s: Krt5 expressing AEC2s possess a distinct gene expression profile enriched in cell proliferation, epithelial development, lung morphogenesis, developmental process, regulation of hemopoiesis genes. While immune system process, cytokine activity, leukocyte activation, cell chemotaxis and chemokine activity genes expression are decreased in Krt5^+^AEC2s

**Additional file 4: Fig. S4**. RNAscope analysis of Krt5: expressing AEC2s and Axin2^+^ AEC2s. High-magnification images of the RNAscope staining of Sftpc, Krt5 and Axin2 of mice lung post CTGF administration. Scale bar, 50 μm

**Additional file 5: Table S1**. Overall clinical characteristics of patients with ARDS

**Additional file 6: Data file S1**. The results of protein mass spectrometry of lung tissue post LPS-induced ALI

**Additional file 7: Data file S2**. The transcriptomic profiles of AEC2s post ALI

## Data Availability

The datasets used for analysis during the current study are available from the corresponding author upon reasonable request.

## Abbreviations

AEC2s: Alveolar type 2 epithelial cells
ALI: Acute lung injury
ARDS: Acute respiratory distress syndrome
CTGF: Connective tissue growth factor
LRP6: LDL receptor-related protein 6
Krt5: Keratin 5
