# Supplementary figures and images for: CTGF promotes the repair and regeneration of alveoli after acute lung injury by promoting the proliferation of subpopulation of AEC2s
Source: Respir Res. 2023 Sep 23;24:227. doi: 10.1186/s12931-023-02512-4 (PMC10517460; doi:10.1186/s12931-023-02512-4)

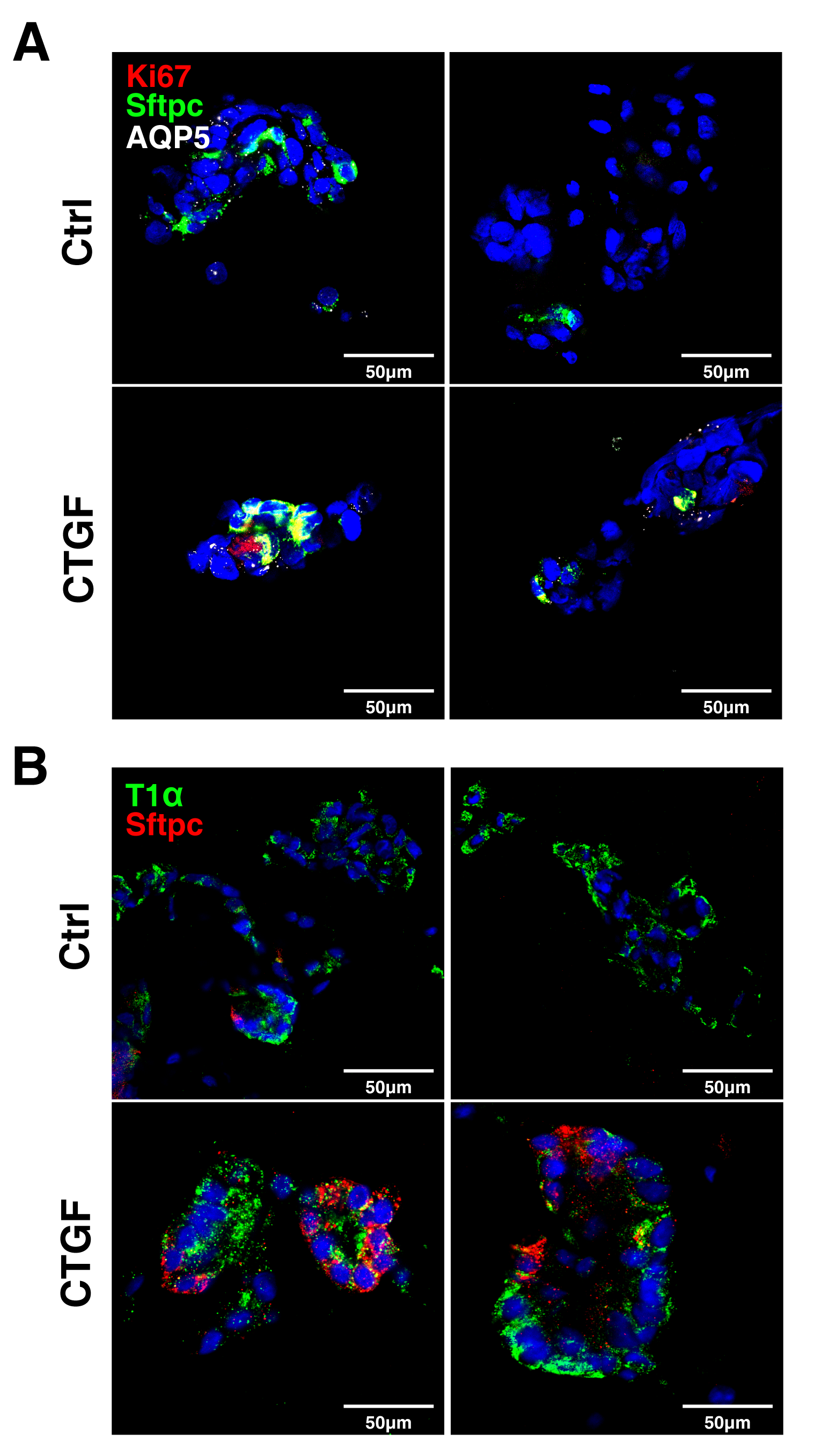

Supplement: Supplementary file 1 — Additional file 1: Fig. S1. CTGF enhance the growth of AEC2s in vitro. (A) High-magnification images of the RNAscope staining of Sftpc, AQP5 and Ki67 of the AEC2s organoids. Scale bar, 50 μm. (B) High-magnification images of the immunofluorescence staining of Sftpc and T1a of the AEC2s organoids. Scale bar, 50 μm [file 12931_2023_2512_MOESM1_ESM.png]

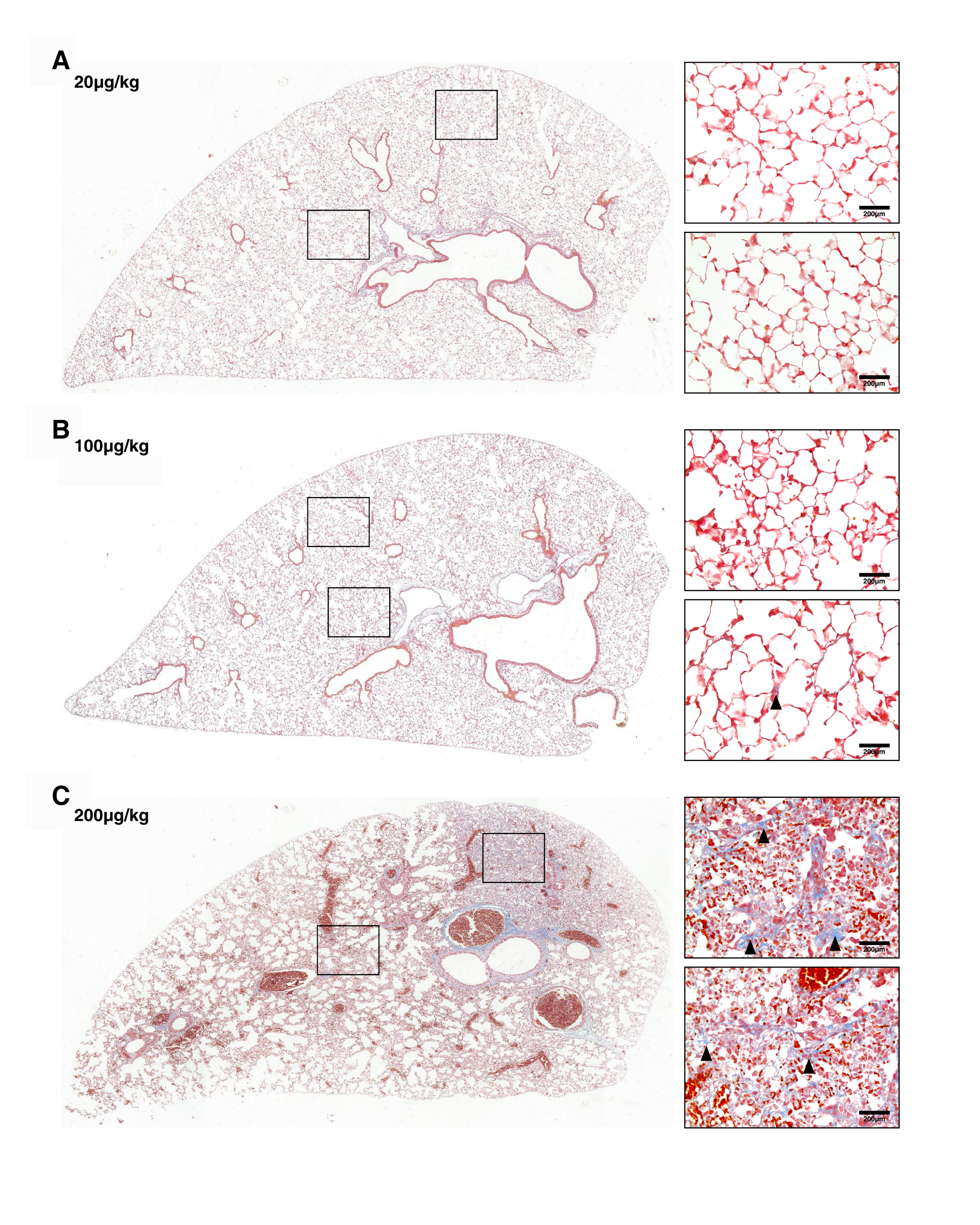

Supplement: Supplementary file 2 — Additional file 2: Fig. S2. Dose effect of CTGF and mouse pulmonary fibrosis. (A) Masson staining was observed in lung tissue after 7 days of continuous administration of 20 ug/kg CTGF, no obvious fiber staining was found in lung tissue. (B) After continuous administration of 100 ug/kg CTGF for 7 days, there was a small amount of fiber deposition in the alveolar septum (black arrow indicates fiber deposition). (C) Significant pulmonary fibrosis was observed after 7 days of continuous administration of 200ug/kg CTGF. Scale bar, 200 μm [file 12931_2023_2512_MOESM2_ESM.png]

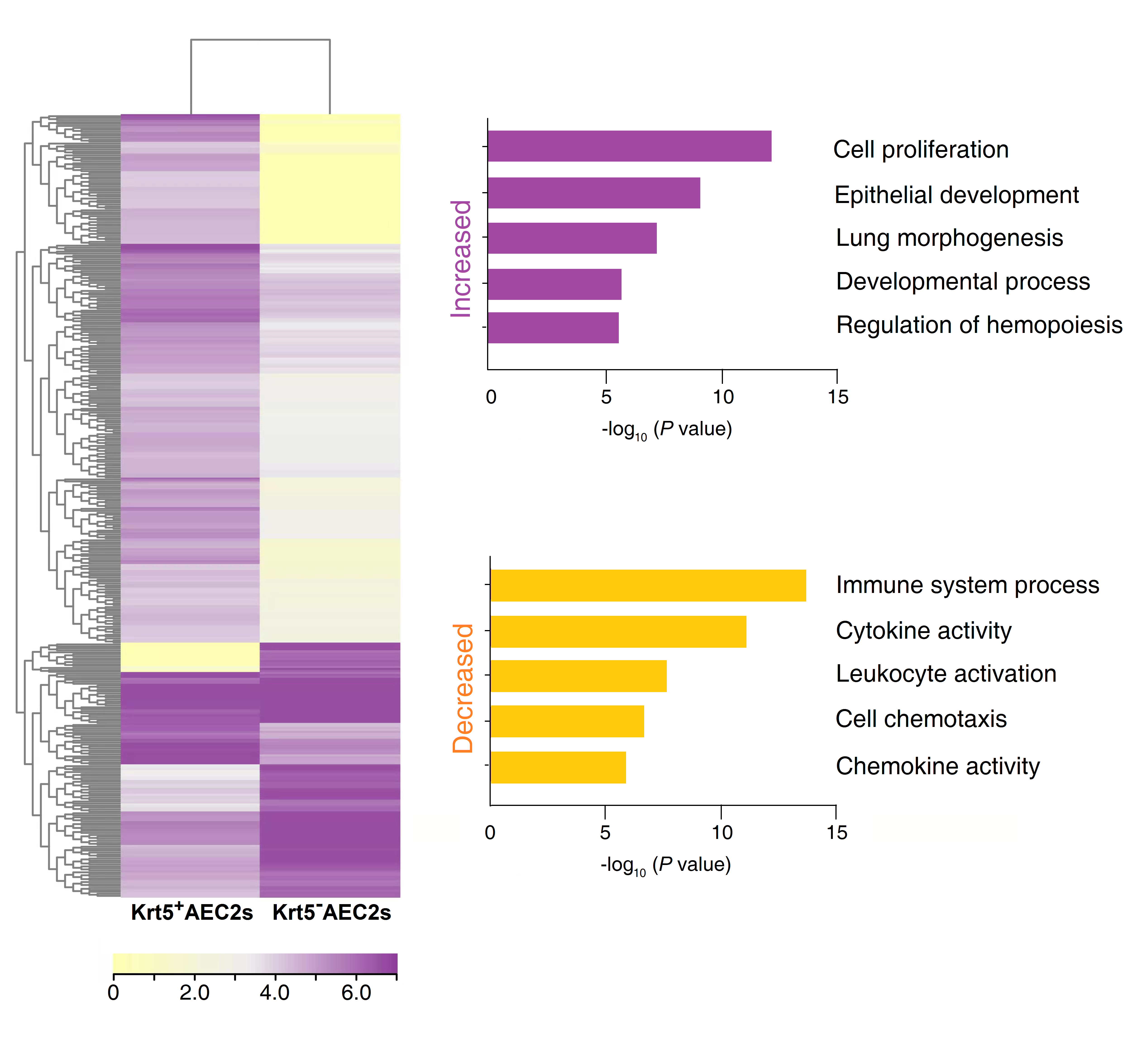

Supplement: Supplementary file 3 — Additional file 3: Fig. S3. RNA-seq analysis of Krt5 expressing AEC2s and Krt5− AEC2s: Krt5 expressing AEC2s possess a distinct gene expression profile enriched in cell proliferation, epithelial development, lung morphogenesis, developmental process, regulation of hemopoiesis genes. While immune system process, cytokine activity, leukocyte activation, cell chemotaxis and chemokine activity genes expression are decreased in Krt5+AEC2s [file 12931_2023_2512_MOESM3_ESM.png]

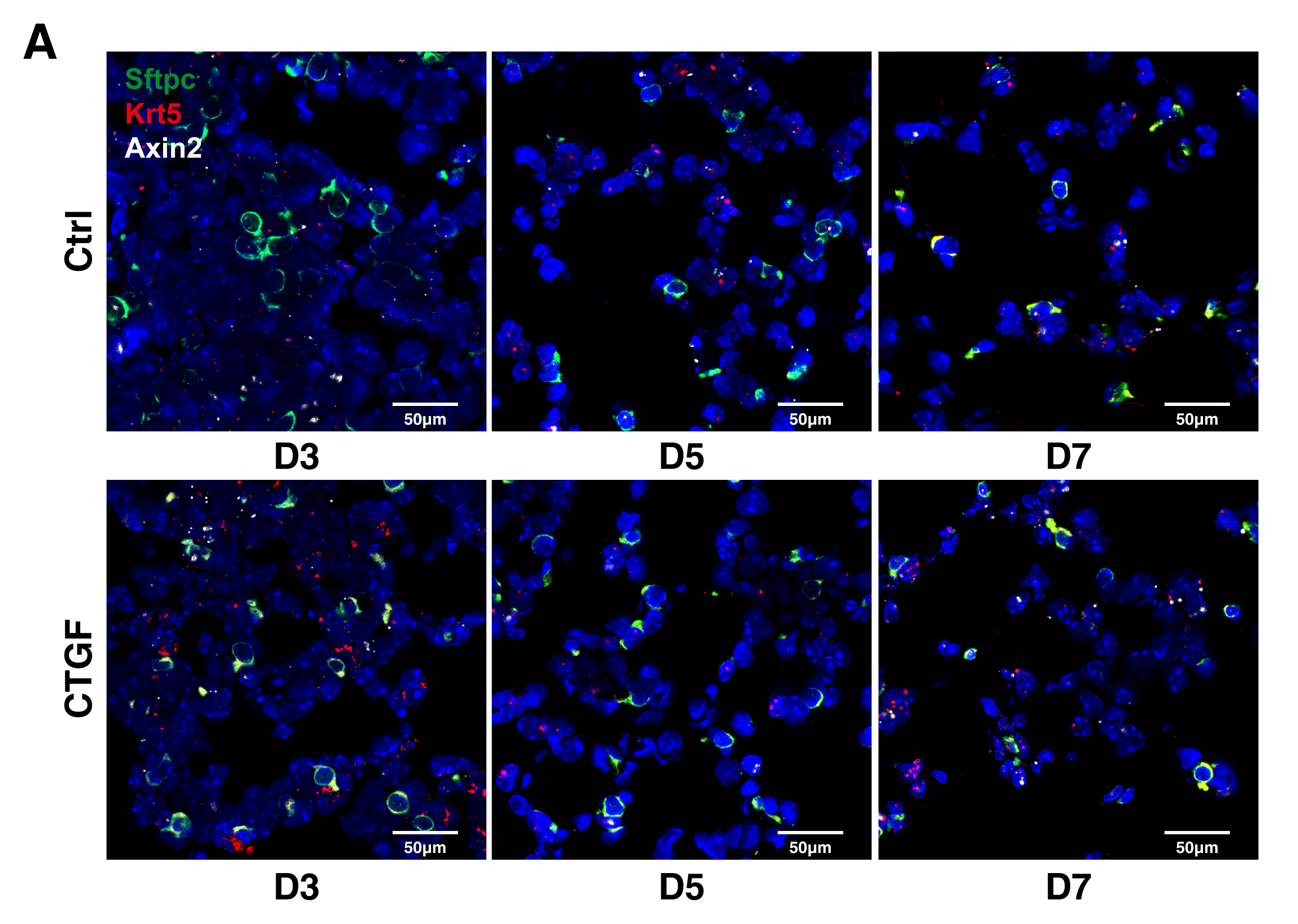

Supplement: Supplementary file 4 — Additional file 4: Fig. S4. RNAscope analysis of Krt5: expressing AEC2s and Axin2+ AEC2s. High-magnification images of the RNAscope staining of Sftpc, Krt5 and Axin2 of mice lung post CTGF administration. Scale bar, 50 μm [file 12931_2023_2512_MOESM4_ESM.png]
