# Supplementary material for: CTGF promotes the repair and regeneration of alveoli after acute lung injury by promoting the proliferation of subpopulation of AEC2s
Source: Respir Res. 2023 Sep 23;24:227. doi: 10.1186/s12931-023-02512-4 (PMC10517460; doi:10.1186/s12931-023-02512-4)
Supplement: Supplementary file 5 — Additional file 5: Table S1. Overall clinical characteristics of patients with ARDS [file 12931_2023_2512_MOESM5_ESM.docx]

**Table S1. Overall clinical characteristics of patients with ARDS**

| Group (ARDS patients, n = 30) | Mild (n = 12) | Moderate (n = 10) | Severe (n = 8) |
| --- | --- | --- | --- |
| Age, years | 51.36 ± 11.27 | 49.18 ± 10.15 | 52.82 ± 15.76 |
| Sex, male/female | 9/3 | 7/3 | 6/2 |
| PaO_2_/FiO_2_, Mean ± SD | 267 ± 31 | 152 ± 38 | 82 ± 12 |
| Timing of diagnosis of ARDS |  |  |  |
| First day | 6 (50.0%) | 6 (60.0%) | 7 (87.5%) |
| Second day | 3 (25.0%) | 2 (20.0%) | 1 (12.5%) |
| Third day | 2 (16.7%) | 1 (10.0%) | 0 (0) |
| Fourth day | 1 (8.3%) | 1 (10.0%) | 0 (0) |
| Length of ICU stay, d | 5 ± 3 | 8 ± 7 | 10 ± 4 |
| Length of ventilation, d | 2 ± 2 | 6 ± 3 | 7 ± 3 |
| Length of hospital stay, d | 7 ± 4 | 14 ± 6 | 17 ± 5 |
| Clinical outcomes |  |  |  |
| Hospital mortality | 2 (16.7%) | 3 (30.0%) | 3 (37.5%) |
